# Comparison of the obstetric anesthesia activity index with total delivery numbers as a single denominator of workload demand in Israeli maternity units

**DOI:** 10.1186/2045-4015-1-48

**Published:** 2012-12-14

**Authors:** Yehuda Ginosar, Alex Ioscovich, Charles Weissman, Ronit Calderon-Margalit, Carolyn F Weiniger

**Affiliations:** 1 Department of Anesthesiology and Critical Care Medicine, Hadassah Hebrew University Medical Center, Jerusalem, Israel; 2 Department of Anesthesiology, Shaare Zedek Medical Center, Jerusalem, Israel; 3 Hebrew University, Hadassah School of Public Health, Jerusalem, Israel

**Keywords:** Activity, Delivery, Workload

## Abstract

**Background:**

Obstetric anesthesia workload demand in Israel has increased due to both an increase in the requests for labor analgesia and a marked increase in the cesarean delivery rate. We propose a new workload-driven performance indicator, the Obstetric Anesthesia Activity Index (OAAI), to serve as a single denominator of obstetric anesthesia activity to enable direct comparison of different hospitals despite dissimilar rates of epidural labor analgesia and cesarean delivery.

**Methods:**

We performed a secondary analysis of two recent national surveys by the Israel Association of Obstetric Anesthesia. In 2005 and 2007 questionnaires were sent to all Israeli hospitals requesting information on the total numbers of deliveries, epidurals, and cesareans annually, together with the anesthesia workforce allocated for the provision of obstetric anesthesia services. The OAAI was calculated based on the premise that epidurals and cesareans are the predominant determinants of obstetric anesthesia workload and that a typical epidural takes about half the time of a typical cesarean. Accordingly, the OAAI for each hospital was calculated as ((0.75 * number of epidurals per year) + (1.5 * number of cesareans per year))/365.

**Results:**

This secondary analysis assessed the 25 maternity units in Israel that participated in both the 2005 and 2007 surveys. As expected, there was a wide inter-hospital variability in epidural and cesarean rates. Hospital rankings based on annual delivery numbers were different from those based on the OAAI. The OAAI correlated closely both with the number of epidurals (2005: Pearson 0.97, *p* < 0.0001; 2007: Pearson 0.97, *p* < 0.0001) and cesareans (2005: Pearson 0.94, *p* < 0.0001; 2007: Pearson 0.92, *p* < 0.0001). These correlations were better for the OAAI than for the annual delivery numbers.

**Conclusions:**

As there was such a wide range of demand for different obstetric anesthesia services among different hospitals, the total number of deliveries is a poor summary indicator of obstetric anesthesia workload. The calculated OAAI better reflected the obstetric anesthesia workload as a single denominator of activity.

## Background

Israel is currently experiencing an anesthesia workforce crisis [1]. The imbalance between anesthesia service demand and supply results from both reduced workforce supply (due to poor recruitment of medical graduates to the specialty and dwindling immigration) and an increase in overall workload demand (particularly in obstetric anesthesia) [1]. Obstetric anesthesia workload in Israel has increased due to both an increase in the cesarean delivery rate (rising from 9.6% in 1992 [2] to 17–18% by 2004 [3]) and epidural labor analgesia. Laboring women may be disproportionately affected by the anesthesia workforce crisis, as labor analgesia may be perceived to be neither life-saving nor profitable [4].

Recently we published a nationwide survey of all obstetric anesthesia unit directors in Israel [4] to assess workload demand and workforce supply in obstetric anesthesia. The number of deliveries per hospital per year is the traditional benchmark for maternity services activity, but this was not the only factor found to determine demands for obstetric anesthesia workload. Our data revealed a remarkably wide variation in the rates of epidural labor analgesia and cesarean delivery among individual hospitals, suggesting that assessment of obstetric anesthesia workforce should not be based purely on the annual number of deliveries.

We aim to present a new tool for determining the demand for obstetric anesthesia personnel. For this purpose, we developed a single composite denominator of obstetric anesthesia activity, the Obstetric Anesthesia Activity Index (OAAI), and performed a secondary analysis of data from our previous study [4] in order to assess its usefulness. We hypothesized that the OAAI would enable direct comparison of obstetric anesthesia workload demands of different hospitals despite dissimilar epidural and cesarean rates. Studies of obstetric anesthesia workload typically report annual delivery numbers. To our knowledge, this is the first report of a workload-directed performance indicator of obstetric anesthesia services. The derivation and limitations of this novel index are described.

## Methods

The Israel Association of Obstetric Anesthesia regularly uses surveys as part of an ongoing assessment of national obstetric anesthesia clinical activity. The methodology of these surveys has been previously described [4]. Briefly, a questionnaire was sent on two occasions to all directors of departments of anesthesia or the nominated directors of the obstetric anesthesia units or services in all Israeli hospitals providing labor and delivery services. The questionnaires contained groups of questions relating to the annual number of deliveries, epidural rates, cesarean rates, and the anesthesia staffing for provision of obstetric anesthesia services (day and night). Other questions included the estimated waiting time for epidural labor analgesia and the anesthesia choices for cesarean delivery. A detailed survey (including anesthesia workforce) was performed in 2005; questions regarding annual number of deliveries, epidural rates, and cesarean rates were repeated in the 2007 survey. Questionnaires were mailed at the end of December with a follow-up telephone reminder after one month and again after an additional month if required. The survey requested data relating to obstetric anesthesia services provided between January and December of the completed year. Twenty-five hospitals were surveyed in 2005. In 2007 there were an additional 2 hospitals providing independent obstetric anesthesia services [4]. In the current secondary analysis only data from the 25 hospitals participating in both surveys are presented. There was a 100% response rate from both surveys.

Units were categorized according to whether there was an anesthesiologist dedicated to the labor ward: (1) 24 h per day, seven days per week; (2) day shifts only (on weekdays); or (3) no shifts either day or night. Hospitals were also categorized based on the predominant ethnic or religious demography of the population using their local facilities: (1) a predominantly ultra-Orthodox Jewish population, (2) a predominantly Bedouin or Arab population, or (3) a heterogeneous population.

As the rates of epidural labor analgesia and cesarean delivery varied widely between the different hospitals for both surveys [4], we sought to develop a single denominator of obstetric anesthesia activity to offset this heterogeneity.

### The obstetric anesthesia activity index (OAAI)

The majority of anesthesia workload in the labor ward comprises epidural labor analgesia and cesarean delivery. The OAAI is a formula composite comprising data taken from the annual numbers of epidurals and cesareans in each institution. In this study, these data were self-reported by the local unit director for each individual institution and were not corroborated by independent observers. Calculation of the OAAI was based on clinical experience that a typical epidural will take approximately half the time of a typical cesarean [5].

Consequently, the OAAI was calculated using the following formula:

(1) $$\begin{array}{r} \mathrm{OAAI}=[\left(\mathrm{no}.\mathrm{of}\;\mathrm{epidurals}\;\mathrm{per}\;\mathrm{yr}*0.75\right)\\ \;+\left(\mathrm{no}.\mathrm{of}\;\mathrm{cesareans}\;\mathrm{per}\;\mathrm{yr}*1.5\right)]/365 \end{array}$$

The ratio of the epidural and cesarean components of the OAAI (OAAI _EPI_ and OAAI _CD_) was also calculated as follows:

(2) $$\begin{array}{rl} \mathrm{OAA}I_{\mathrm{CD}/\mathrm{EPI}}= & \left(\mathrm{no}.\mathrm{of}\;\mathrm{cesareans}\;\mathrm{per}\;\mathrm{yr}*1.5\right)\\ & /\left(\mathrm{no}.\mathrm{of}\;\mathrm{epidurals}\;\mathrm{per}\;\mathrm{yr}*0.75\right) \end{array}$$

### Statistical methods

Quantitative variables are presented as mean ± standard deviation and were compared between groups using the independent samples t-test. Categorical data are presented as percentages and were compared between the study groups using the chi-square test or Fisher's exact test. The correlation between two variables was compared using Pearson's correlation coefficient where appropriate (variables are continuous and normally distributed; the two variables were independent and the relationship between them was linear). All statistical tests were two sided and a *p*-value < 0.05 was considered statistically significant. Statistical analysis was performed using SPSS 17.0 (SPSS Inc. Chicago, Illinois).

## Results

**Figure 1:**
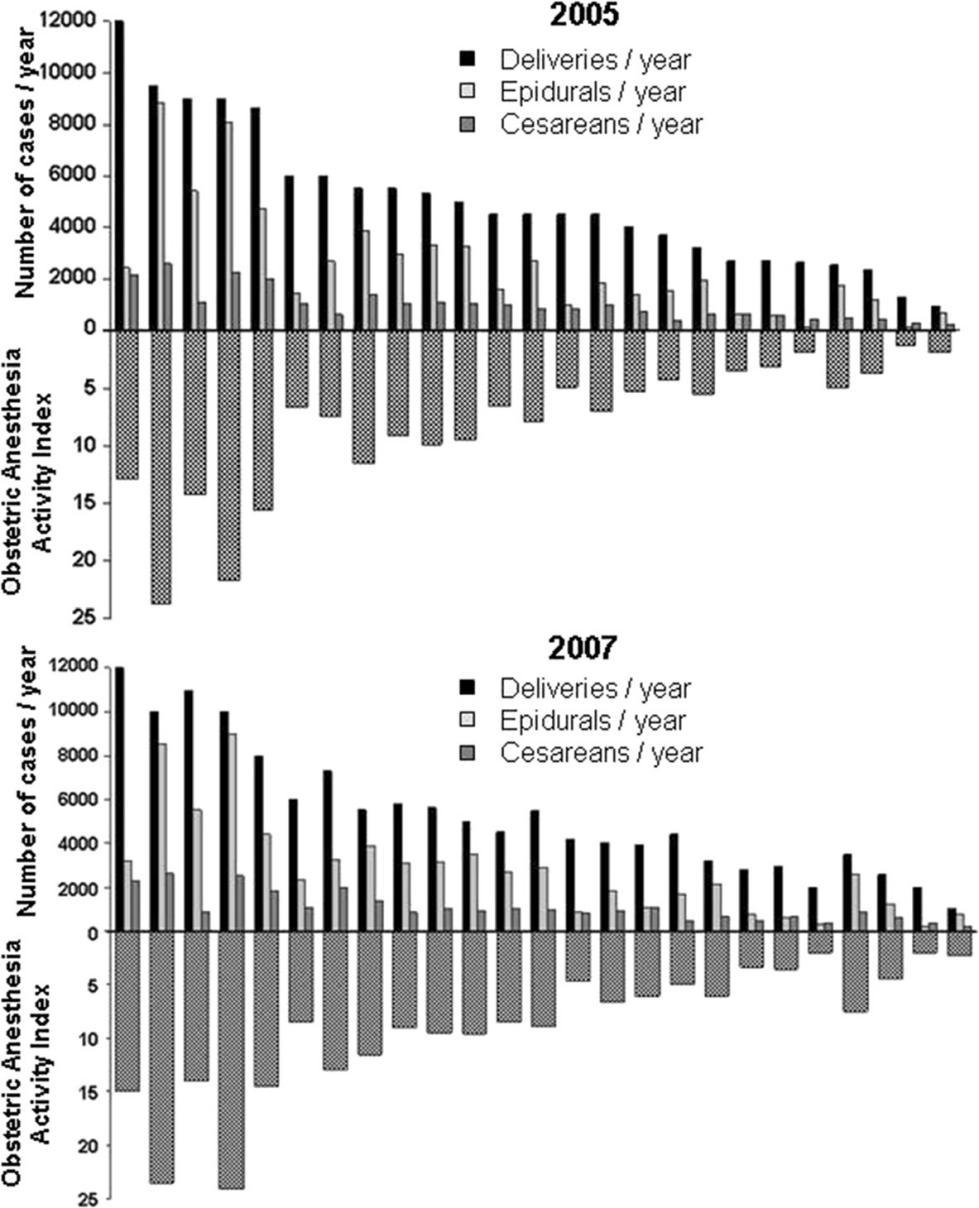
**The wide variation in epidural and cesarean delivery rates in Israeli hospitals makes annual delivery numbers a poor assessment of obstetric anesthesia activity.** For each composite figure, the upper portion consists of annual numbers of deliveries, epidurals, and cesareans. The lower portion represents the calculated OAAI. Ranking of hospital activity by annual delivery numbers alone does not reflect the ranking by OAAI. Data for 2005 (upper) and 2007 (lower).

**Figure 2:**
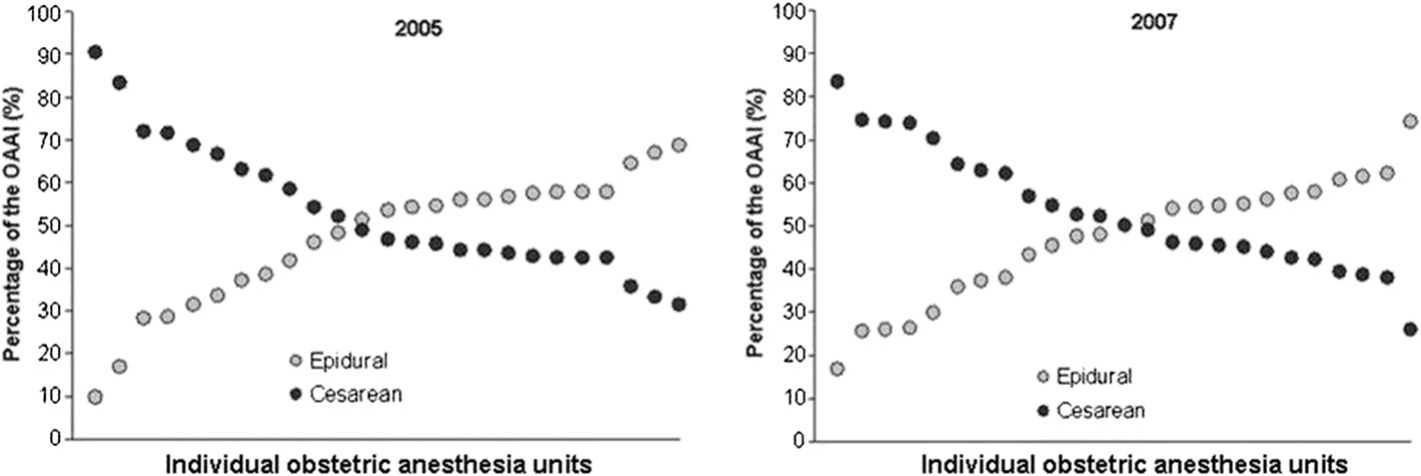
**The wide variation in epidural and cesarean delivery rates in Israeli hospitals is reflected in the contributions of epidural analgesia and cesarean anesthesia to the total OAAI in individual hospitals.** Data for 2005 (left) and 2007 (right).

There was a wide inter-hospital variability in the epidural and cesarean rates in both periods surveyed (Table 1). The annual delivery numbers do not reliably reflect these key components of obstetric anesthesia workload. Hospital rankings based on annual delivery numbers were significantly different from rankings based on the OAAI (Figure 1). There was a wide inter-hospital variation for the OAAI_CD/EPI_ because of a wide variation in epidural and cesarean components of OAAI (Figure 2). In 2005, median OAAI_CD/EPI_ was 0.88; range 0.45 (60% epidural, 12% cesarean) to 9.5 (4% epidural, 16% cesarean). In 2007, median OAAI _CD/EPI_ was 1.0; range from 0.35 (50% epidural, 8% cesarean) to 5.0 (10% epidural, 20% cesarean).

**Table 1:** Raw data from national surveys of obstetric anesthesia units in Israel, showing the annual delivery numbers and the epidural and cesarean delivery rates (%) with the calculated OAAI

**2005**				**2007**			
**Deliveries (no./year)**	**Epidural rate (%)**	**Cesarean rate (%)**	**OAAI**	**Deliveries (no./year)**	**Epidural rate (%)**	**Cesarean rate (%)**	**OAAI**
12,000	20	18	12.92	12,000	27	19	14.93
9,500	93	27	23.79	10,000	85	26	23.61
9,000	60	12	14.20	11,000	50	8	14.01
9,000	90	25	21.73	10,000	90	25	24.14
8,600	55	23	15.61	8,000	55	23	14.52
6,000	24	17	6.65	6,000	40	18	8.44
6,000	45	10	7.46	7,300	45	27	13.03
5,500	70	25	11.58	5,500	70	25	11.58
5,500	54	19	9.16	5,782	54	15	9.02
5,300	63	20	9.84	5,600	56	18	9.43
5,000	65	20	9.45	5,000	70	18	9.60
4,500	35	22	6.59	4,500	60	23	8.53
4,500	60	18	7.88	5,440	53	18	8.87
4,500	22	18	4.91	4,200	20	19	4.68
4,500	40	22	6.95	4,000	45	23	6.60
4,000	35	17	5.18	3,900	27	28	6.05
3,700	40	10	4.26	4,398	38	10	4.96
3.200	60	18	5.60	3,188	67	20	6.10
2,700	22	22	3.39	2,788	26	18	3.28
2,690	20	20	3.10	2,969	20	22	3.56
2,600	4	16	1.89	2,000	15	18	1.98
2,500	68	20	4.85	3,500	74	24	7.50
2,350	50	18	3.72	2,548	49	23	4.36
1,300	10	20	1.28	2,000	10	20	1.97
900	70	23	1.85	1,000	75	24	2.16

**Figure 3:**
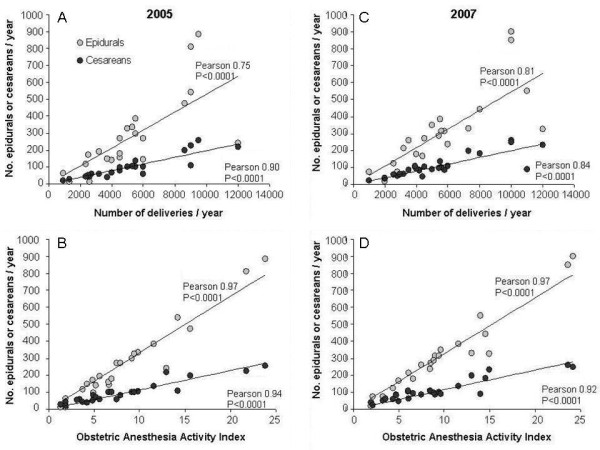
**Correlation of annual epidural and cesarean numbers with annual delivery numbers (A, C) and with OAAI (B, D).** Data for 2005 (**A**, **B**) and 2007 (**C**, **D**). The OAAI correlated more closely with both the number of cesarean deliveries and the number of epidurals. Although coupling exists as the OAAI is derived from both cesarean delivery and epidural rates, it is precisely for this reason that a single denominator is a more useful measure of obstetric anesthesia activity than annual delivery numbers.

**Figure 4:**
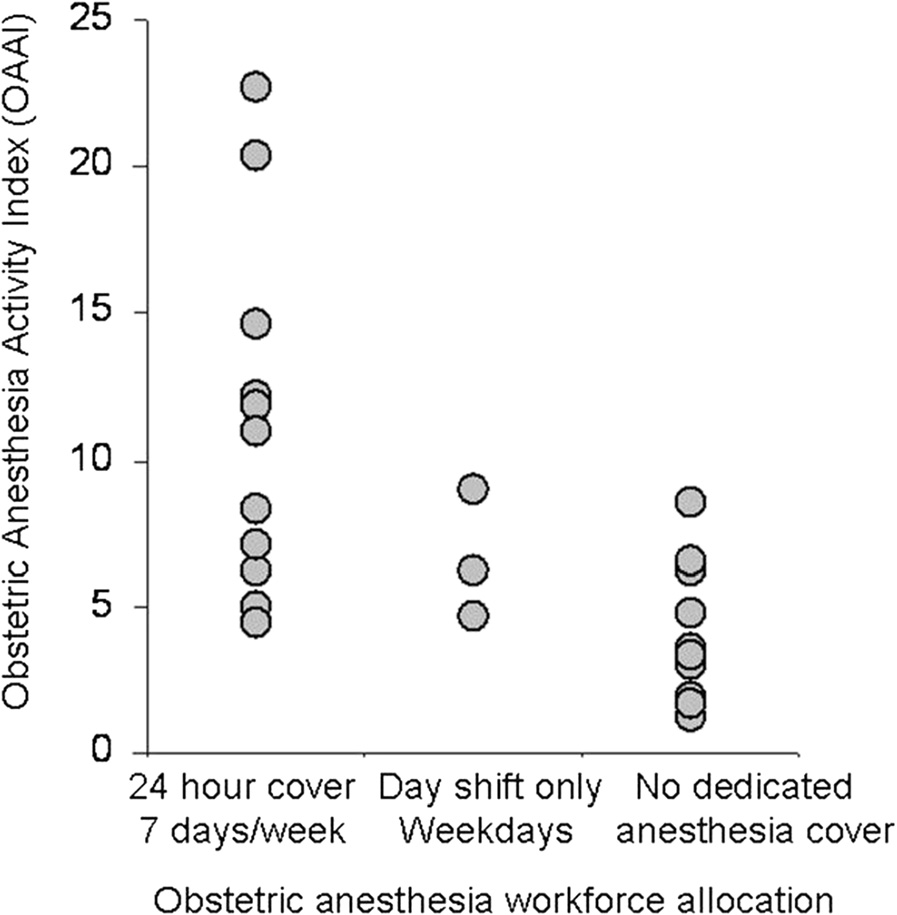
The OAAI for individual hospitals according to obstetric anesthesia workforce allocation (data for 2005 only; 2007 survey did not collect workforce data).

The OAAI correlated with epidural rates and cesarean rates more closely than did the annual number of deliveries (Figure 3). There was no clear relationship between the anesthesia workforce allocation to the labor ward and the obstetric anesthesia workload as measured by the OAAI (Figure 4).

## Discussion and conclusion

This study reports data derived from a self-reported questionnaire from obstetric anesthesia unit directors. The study found that total number of deliveries was a poor measure of obstetric anesthesia workload. The OAAI is a workload-directed performance indicator and better reflects the obstetric anesthesia workload than merely measuring the total number of deliveries. Use of the annual number of deliveries as the bench-mark comparator for maternity services will under-estimate obstetric anesthesia activity in centers with high epidural rates and will over-estimate it in centers with low epidural rates. Consequently, the OAAI may be useful as a denominator in obstetric anesthesia workforce staffing calculations.

There was a wide range of demand for different obstetric anesthesia services among the different hospitals. However, there was no clear relationship between the allocation of obstetric anesthesia workforce to labor wards in Israel and the obstetric anesthesia workload as measured by the OAAI.

Ensuring adequate staffing levels for obstetric anesthesia units is important for both patient satisfaction and patient safety. From our previously published data, hospitals with a dedicated anesthesiologist in the labor ward 24 h per day/7 days per week had a two-fold increase in the epidural rate and half the epidural waiting time, when compared to hospitals where the anesthesiologist had to be called from the operating room [4]. In addition to the provision of analgesia, a functioning epidural catheter can be used for the provision of epidural surgical anesthesia for urgent cesarean delivery without the need for potentially hazardous emergency general anesthesia. Maternal death due to anesthesia is the sixth leading cause of pregnancy-related death in the United States [6] and most anesthesia-related deaths occur during general anesthesia for urgent cesarean delivery. The risk of maternal death from complications of general anesthesia is 17 times that associated with regional anesthesia [7]. Therefore, the finding in our earlier study that there was an inverse relationship between the epidural rate for labor analgesia and the choice of general anesthesia for emergency cesarean delivery [4] suggests that inadequate obstetric anesthesia workforce supply may have adverse effects on patient safety. Other studies have reported the adequacy or inadequacy of obstetric anesthesia workforce in relation to total delivery numbers [8]. The current study is the first stage in an approach that will attempt to define the adequacy of obstetric anesthesia workforce in relation to activity.

The epidural component of the OAAI includes time taken for pre-analgesia assessment, sterile preparation, block placement, incremental drug dosing (over several minutes), and at least 15-20 min bedside observation following completion of drug administration. A labor epidural should never take less than 30 min, and typically takes in the region of 45 min. The time spent on anesthesia for cesarean delivery is rarely less than 90 min although surgical time varies between hospitals, surgeons, and patient risk-factors, and time spent waiting for post-anesthesia care unit.

Like any composite measure, the OAAI does not specifically identify the individual predominant contributing component. The OAAI ignores requests to provide supplementary epidural analgesia throughout labor [9], although this element can be greatly reduced by the use of patient-controlled analgesia pumps. The OAAI ignores clinical activities other than epidural analgesia and cesarean anesthesia (including anesthesia for retained placenta and complicated vaginal deliveries, antenatal or pre-operative consultation, and resident training). In some centers, the obstetric anesthesia team also provides anesthesia services for non-obstetric gynecological operations and for post-anesthesia care units. The OAAI cannot account for lengthy epidural analgesia and cesarean deliveries or differentiate between day/night/weekend shifts and experience of personnel. Accordingly, the OAAI is not a measure of the total activity of the obstetric anesthesia services.

Based on these limitations, it is important to appreciate that although the OAAI is numerically identical to the time (in hours) spent engaged in epidurals and cesareans in an average 24 h period, the OAAI is a dimensionless index of activity and is not a measurement of time.

Additionally, the OAAI does not consider the degree of workforce redundancy that is required to safely accommodate extra workload during peak activity or provide expert back-up when the maternity services are located in remote locations away from the main anesthesia services. Provision of back-up is particularly important when considering emergency cesareans and the data could not differentiate between elective and emergency cesarean delivery. Finally, while it is obvious that coupling exists, as the OAAI is derived from both epidural rates and cesarean rates, it is precisely for this reason that this single denominator is a more reliable measure of activity than annual delivery numbers.

A limitation of the data upon which this secondary analysis is based is that data were self-reported and not corroborated; in almost all cases data were approximated by the unit directors. Part of the explanation for this finding is that many hospitals have no computerized data management system. A national observational study is underway in Israel to assess the obstetric anesthesia workforce supply and work load demand ratio, based on the OAAI, and to correlate this with quantifiable measures of adequacy of obstetric anesthesia services. That study may provide corroboration for the data presented in this study and will attempt to identify an ideal obstetric anesthesia staffing number based on the OAAI.

In summary, the use of the OAAI may facilitate a comparison of the workforce supply – workload demand ratio (and defined obstetric outcomes) for hospitals with different geographical (center versus periphery) and cultural (ultra-Orthodox Jews, Arabs and Bedouins, versus heterogeneous) demographic populations. Such studies may provide the data to support a change in health care resource allocation, to provide obstetric anesthesia workforce commensurate with obstetric anesthesia workload demands [10], and to provide uniform levels of care throughout the country. Based on these studies, it is possible that future recommendations for obstetric anesthesia staffing ratios will need to use the OAAI, or a similar index, as a single workload denominator.

## Competing interests

There are no competing interests declared for any author.

## Authors’ contributions

YG devised the obstetric anesthesia activity index; YG and CFW wrote the draft manuscript; AI, CW and RCM all read the draft manuscript and all made important intellectual contributions to the final version.

## Authors’ information

Yehuda Ginosar is Senior Lecturer of Anesthesia and Director of the Mother and Child Anesthesia Unit at the Hadassah–Hebrew University Medical Center. He is a past Chair of the Israel Association of Obstetric Anesthesia. His main research interests are focused on anesthetic interventions to improve fetal well-being in high risk pregnancy and spinal cord anesthetic pharmacology.

Alex Ioscovich is Clinical Senior Lecturer of Anesthesia and Director of the Obstetric Anesthesia Unit at Shaarei Zedek Medical Center. He is the current Chair of the Israel Association of Obstetric Anesthesia. His main clinical research interests are focused on clinical obstetric anesthesia and grandmultiparity.

Charles Weissman is Professor of Anesthesia and Director of the Department of Anesthesiology and Critical Care, at the Hadassah–Hebrew University Medical Center. His main research interests are focused on critical care, nutrition, surgical stress response, and the organization of anesthesia services.

Ronit Calderon-Margalit is Senior Lecturer in Epidemiology at the Hebrew University–Hadassah, Braun School of Public Health. Her main research interests are focused on women's health and perinatal care.

Carolyn F. Weiniger is Senior Lecturer in Anesthesiology and lead clinician in the Antenatal Anesthesia Consultation Service at the Hadassah–Hebrew University Medical Center. Her main research interests are focused on major obstetric hemorrhage, the use of spinal anesthesia for breech conversion, the use of an ultra-short-acting opioid analgesic drug as an alternative to epidural for labor pain relief, and the development of long-acting local anesthetic polymers.
